# Association between leisure-time physical activity and the built environment in China: Empirical evidence from an accelerometer and GPS-based fitness app

**DOI:** 10.1371/journal.pone.0260570

**Published:** 2021-12-31

**Authors:** Long Chen, Zhaoxi Zhang, Ying Long

**Affiliations:** 1 School of Architecture, Tsinghua University, Beijing, China; 2 Department of Environmental Science, Aarhus University, Roskilde, Denmark; 3 Danish Big Data Centre for Environment and Health (BERTHA), Aarhus University, Aarhus, Denmark; 4 Hang Lung Center for Real Estate, Tsinghua University, Beijing, China; 5 Key Laboratory of Eco Planning & Green Building, Ministry of Education, Beijing, China; Xiamen University, CHINA

## Abstract

To reexamine the relationship between leisure-time physical activity (LTPA) and the built environment (BE), this paper takes advantage of the massive amount of data collected by an accelerometer and GPS-based fitness mobile app. Massive LTPA data from more than 3 million users were recorded by Codoon in 500m by 500m grid cells and aggregated to 742 natural cities in mainland China. Six BE indicators were quantified using GIS at the city scale. Robust regression analysis was used to estimate the correlation between LTPA and BE. Five of six BE indicators—connectivity, road density, land use mix, points of interest density, and density of parks and squares—were significantly, positively, independently, and linearly related to LTPA in the regression analysis. The study obtains findings that are consistent with the previous literature but also provides novel insights into the important role of POI density in encouraging LTPA, as well as how the relationship between LTPA and BE varies by time of day. The study also sheds light on the embrace of new technology and new data in public health and urban studies.

## Introduction

The pandemic of physical inactivity has become a global issue. The WHO recommends engaging in physical activity (PA) as an important, modifiable behavior for preventing noncommunicable chronic diseases, such as obesity, diabetes, and cardiovascular disease, and for promoting public health. While many studies have explored ways to encourage PA through education [1], social support [2], and health promotion strategies [3], an increasing number of studies in both urban planning and public health have begun to examine the socioecological context of PA, suggesting that the environment in which PA takes place should be considered. The accumulation of evidence demonstrates that people who live in walkable neighborhoods, which are characterized by higher population density, a mix of different urban functions, interconnected street networks, and access to shops and services, public transport, parks and recreational facilities, tend to be more physically active [4–6]. When considering the factors that are associated with PA, knowing the built environment (BE) becomes essential, as it can be altered by evidence-based planning and design to encourage positive engagement in PA [7].

However, the existing evidence on the relationship between PA and BE remains limited. First, most existing literature are from Western countries and Latin America, such as studies in the U.S. [8], Australia [9], and Brazil [10]; such focus lacks perspective from Asian cultural contexts and overlooks population heterogeneity. Second, due to the insufficiency of administration and labor in research, self-report measures are commonly used in assessing PA [11, 12]. For example, the International Physical Activity Questionnaire (IPAQ), which originated in Geneva in 1998, provides a set of preestablished questions that can be used internationally to obtain comparable estimates of PA and has become a widely accepted tool for PA assessment. The IPAQ collects PA data by asking participants to recall their vigorous and moderate activities during the last 7 days. However, this conventional method relies heavily on participants’ motivation and input, thereby not only limiting the number and socioeconomic features of participants but also resulting in subjective bias that hampers accuracy.

Advancements in technology such as the development of low-cost sensors, wearable devices, cloud computing, and artificial intelligence enable the application of wearable accelerometers that offer advantages over self-reported measures of PA. Wearable accelerometers can record the intensity, rate, and duration of the activity, and their reliability and validity have already been extensively discussed and verified [13]. Although such advanced technologies improve the accuracy of observation, the high costs of wearable devices have usually resulted in smaller sample size, making it challenging to apply this method for a large-scale analysis [14]. Moreover, both the IPAQ and the wearable accelerometer have their own shortcomings. Nevertheless, the proliferation of smartphones is enabling massive and consecutive long-term tracking through applications that utilize smartphones’ built-in GPS and accelerometers, such as fitness apps (MyFitnessPal, Codoon, JEFIT). It enables us to achieve a massive amount of data in human activities at much finer scales via data from fitness apps. Specifically, this paper takes advantage of the massive PA data at a fine scale that were collected by Codoon (https://www.codoon.com), which is one of the top fitness apps in China, to reexamine the relationship between PA and BE. Using a sample of more than 3.79 million Codoon app users in Mainland China, which is the largest sample size (to our knowledge) for such research to date, this study empirically aggregated PA data to the city level (across 742 natural cities) to illuminate the massive measurement of PA in China and, further, examined the association between objectively quantified PA and BE.

## Data and method

### Unit of analysis

In the existing literature, the association between PA and BE has mostly been discussed at the micro scale, such as the neighborhood [15–18] and grid cells [4]. PA measurement and sample size are the main reasons that previous studies could only examine a limited number of individuals and their surroundings. Taking advantage of Codoon data for more than 3.7 million users, this study takes the redefined natural city [19] as the unit of analysis to examine the correlation between PA and BE for a large-scale analysis. This study aims to examine how BE at the city scale may affect the average PA level, whereas existing studies focus on the influences of urban design at the neighborhood scale on individual participants.

The redefined natural city [19] has geographic boundaries that are delineated by the density of points of interest (POI). Specifically, a natural city is defined as clusters of human settlements and activities, and it depicts the central urban regions whose boundaries are developing naturally instead of those outlined by administrative purposes. Compared with administrative divisions, the natural city boundary excludes large areas of uninhabited land, such as forest, cropland and wasteland, which generally cause misrepresentation of the BE that may affect PA.

### Outcome variable

This study implements the Codoon app to quantitatively and objectively measure PA for a large sample size at fine scale. Because fitness tracking is the main function of the Codoon app, this study classified the recorded PA data as leisure-time physical activity (LTPA) rather than transport-related physical activity (TRPA). The app enables users to monitor, record and share their LTPA, such as walking, hiking, jogging, cycling, and gym fitness activities. In accordance with the Privacy Policy (www.codoon.com/help/m_license.html), the users have authorized the app to collect personal information and activity logs, as well as the use of anonymized and aggregated data, which enables Codoon to construct a national database of 24-hour PA recorded by the GPS and accelerometer functions embedded in smartphones. Each time a Codoon app user finishes an activity (i.e., LTPA) logged in the app, the Codoon database generates an individual LTPA event that records the time and location of the activity, however, neither the duration nor the type of the activity will be recorded. Codoon then created a raster map of mainland China with 500 m by 500 m grid cells and calculated the total number of the individual LTPA events in each grid cell.

**Fig 1:**
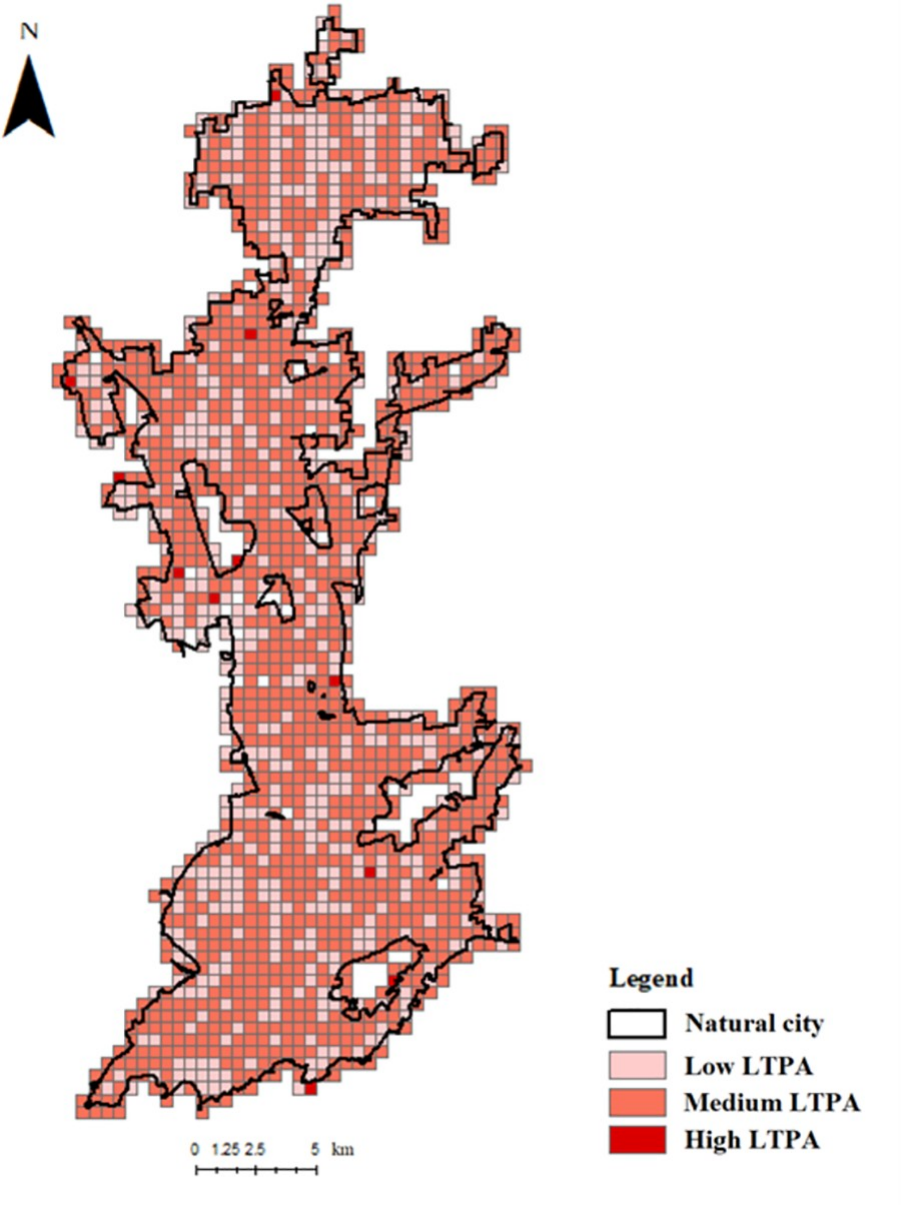
An example of the LTPA data in Qingdao city.

Before releasing the data, Codoon normalized the original LTPA data by defining the total amount of LTPA events in all grid cells as 1, and thus each cell was assigned with a scaled-down number that represents the ratios to the national total. In other words, the normalized data in each grid cell now represents a proxy of the LTPA level (frequency of LTPA recorded by Codoon app), which is comparable among all grid cells. In this study, we achieved the cross-sectional data that recorded the accumulated LTPA events from January to June in 2018 for each grid cell. The grid cells are then geographically aggregated to natural cities to generate the level of LTPA in each city, which was later calculated at per capita basis and used as the dependent variable of the regression models. The LTPA variable is then further classified into two sub-variables based on the time of the activities, as morning LTPA that records activity logs from 6 to 9 a.m., and night LTPA from 6 to 9 p.m. To better illustrate the LTPA data, we randomly selected a city to show how we aggregated the city level LTPA (Fig 1), where the level of LTPA in each cell is categorized using Jenks Natural Breaks.

**Fig 2:**
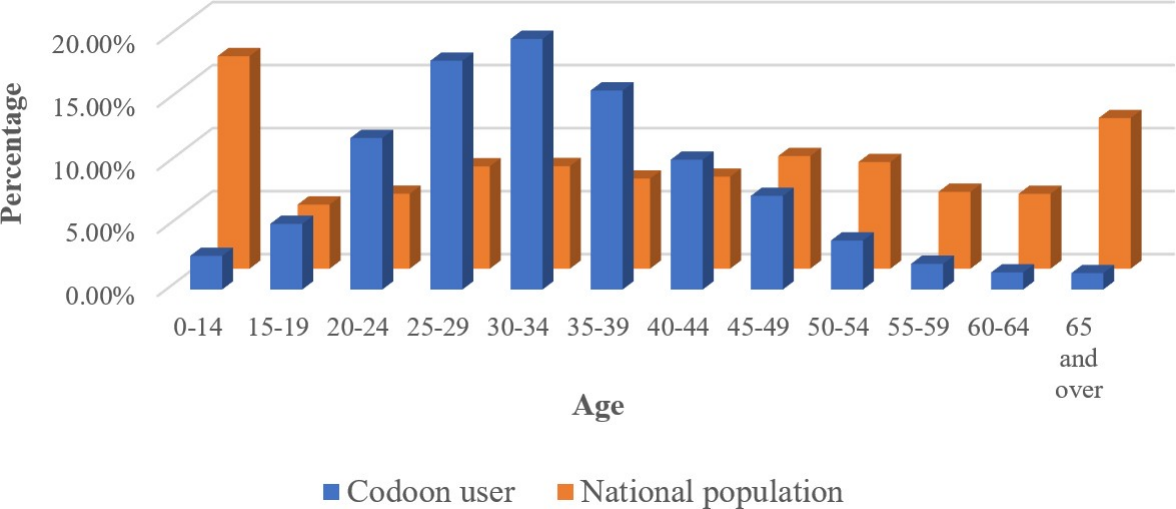
Age composition of Codoon users and the national population in 2018.

For privacy reasons, Codoon users’ basic demographic profiles were anonymized and aggregated at the national level to eliminate any identifying information. Notably, Codoon can only captures the LTPA data of its app users, and it is thus essential to examine these user profiles and evaluate whether the results from this study could be generalized to the general population. Fig 2 compares the age compositions of Codoon users and the national population in 2018, showing that the majority (88.78%) of Codoon users are between ages 15 and 50, as children and elderly people have limited smartphone access. This result also indicates that the mean age of Codoon users is younger than that of the national population, so our study would target the younger generation, aged between 15 and 50. In this age range, the age compositions of Codoon users and the national population show similar distributions (Fig 2). Regarding the gender ratio, the percentages of male in Codoon users and national population are 51.2% and 51.1%, respectively, implying negligible bias from the Codoon data.

### Independent variables

#### BE indicators

The development of geographic information systems (GIS) has enabled public health researchers to objectively quantify BE, especially for studies involving a larger geographic area. Unlike perceived environment measures, which refer to the self-report data on participants’ perceptions of the BE, GIS approaches have become more widely used in studies linking BE with PA [16, 20]. Frequently used variables in measuring BE with GIS data and tools include density [21, 22], land use mix [4, 23], accessibility of recreational facilities [15, 24], and street patterns [4, 15]. In accordance with the existing literature, population density, land use mix, and connectivity remain the main BE indicators in this study. Among which, population density is calculated using the ambient population data from LandScan divided by the total land area of each city. The Entropy Index is used to calculate the land use mix, and 7 categories of POIs, including companies, education, governmental, park, residential, transportation and commercial were employed in the calculation, where higher level of Entropy mean higher level of mix. Density of road junctions is selected as the representing variable for connectivity and is calculated as the number of junctions per sq. km. In addition to those, “POI density”, “road density” and “density of parks and squares” are also included as BE indicators that affect LTPA. We identified a few studies in the literature that have examined these correlations, such has how POI density increases urban vibrancy, and in turn attracts outdoor activities in general [25, 26], and how urban street [27, 28] and parks [29, 30] act as the primary venues for the LTPA.

#### Control variables

Sociodemographic characteristics, neighborhood attributes, and weather conditions are included as the control variables, based on existing studies [15–18] and our systematic thinking. Table 1 lists all the variables introduced.

**Table 1:** A full list of dependent and independent variables.

Name	Description	Data source	Year	
**Dependent variable**				
*LTPA*	Level of LTPA at per capita basis in each city	Codoon app		2018
**Independent variables**				
*Population density*	Number of ambient population per sq. km.	LandScan		2016
*POI density*	Number of POI per sq. km.	Gaode Map		2016
*Land use mix*	Entropy index of POI.	Gaode Map		2016
*Connectivity*	Number of road junctions per sq. km.	Gaode Map		2016
*Road density*	Length of primary and secondary roads per sq. km.	Gaode Map		2016
*Parks and squares*	Area of parks and squares per sq. km.	Areas of Interest product based on Gaode Map		2019
**Control variables**				
*Total population*	Total number of ambient population	LandScan		2016
*Density of stadium*	Number of stadiums per sq. km.	Areas of Interest product based on Gaode Map		2019
*Gender*	Percent of male population (*%*)	China City Statistical Yearbook		2018
*Age group*	Percent of population with age 65 and older	China City Statistical Yearbook		2018
*GDP per capita*	GDP per capita (*yuan*)	China City Statistical Yearbook		2018
*Air quality*	Average PM 2.5 concentration (*ug/m*^*3*^)	China National Environmental Monitoring Centre		2018.01–2018.06
*Wind*	Average wind speed (0.1*m/s*)	National Meteorological Information Center		2018.01–2018.06
*Temperature*	Average air temperature (0.1*°C*)	National Meteorological Information Center		2018.01–2018.06
*Humidity*	Average relative humidity (*%*)	National Meteorological Information Center		2018.01–2018.06
*Night light density*	Number of night light pixels per sq. km.	Luojia 1–01 nighttime light		2018

A list of descriptive statistics of all the variables for 742 cities in the analysis is provided in Table 2.

**Table 2:** Descriptive statistics of all the variables in the regression models.

Variable	Mean	Std. Dev.	Min	Max
*All day LTPA*	34.480	53.399	0.174	620.787
*Morning LTPA*	9.666	16.371	0.046	317.622
*Night LTPA*	4.07	7.769	0.025	102.594
*Population density*	5404.254	3343.576	140.818	31010.76
*Connectivity*	25.394	13.77	4.191	176.541
*Road density*	5716.766	2101.517	1127.795	32598.88
*POI density*	87.938	125.463	4.926	1160.976
*Land use mix*	0.348	0.089	0.062	0.557
*Parks and squares*	0.018	0.034	0	0.471
*Density of stadium*	0.321	0.302	0	2.656
*Total population*	357792	1536421	477	29719252
*Gender*	0.51	0.008	0.473	0.542
*Age group*	0.093	0.019	0.018	0.165
*GDP per capita*	83413.66	37861.79	23855	218837.3
*Air quality*	4.289	0.884	1.94	6.94
*Wind*	2.249	0.702	0.927	6.618
*Temperature*	13.624	3.472	0.091	24.858
*Humidity*	64.973	12.046	29.812	85.265
*Night light density*	1576733	773133	0	9909563

#### Analysis

Since the LTPA data collected by Codoon have time and location stamps to indicate when and where the activities happened, this study analyzes the features of the temporal and spatial distribution of the LTPA data and links the results with the BE variables to identify the potential influencing factors that could encourage or discourage the PA level. Each grid cell in our data has eight attributes indicating LTPA in eight time slots, divided evenly from 0:00 to 24:00, so we adopted time clustering based on the K-means algorithm in SPSS to identify the temporal feature. Regression analysis was conducted in SPSS to model the relationship between LTPA and BE variables to examine the effects of urban spatial characteristics. The results of the cluster analysis show that the periods between 6:00–9:00 and 18:00–21:00 have significant clustering characteristics. While acknowledging the temporal pattern of LTPA, this study specifically examined the correlations between LTPA and BE in three different time periods: all day LTPA from 0:00 to 24:00, morning LTPA from 6:00 to 9:00, and night LTPA from 18:00 to 21:00.

The pairwise correlations were first calculated between the key variables before adding them into the regression models. The results shown in Table 3 indicates potential associations between LTPA and BE variables. For example, population density is found to be negatively correlated with all LTPA variables, while density of parks and squares are showing significant but positive coefficients. However, such correlations need to be further examined in the regression models when all other variables are co-existed.

**Table 3:** The pairwise correlations between LTPA and influencing variables.

Variables	All day LTPA	Morning LTPA	Night LTPA
*Population density*	-0.4522***	-0.4069***	-0.3824***
*POI density*	-0.0984***	-0.0136	-0.1012***
*Connectivity*	-0.0073	-0.0021	0.0679*
*Land use mix*	0.0994***	0.0592	0.0975***
*Road density*	0.0281	0.0514	0.0709*
*Park and square*	0.0772**	0.0878**	0.0997***
*Density of stadium*	-0.0471	0.0231	-0.0616*
*Total population*	-0.2793***	-0.2743***	-0.1817***
*Gender*	-0.0774**	-0.0701*	-0.0285
*Age group*	0.1353***	0.1022***	0.1266***
*GDP per capita*	-0.0378	-0.1101***	0.0339
*Air quality*	0.0646*	0.0929**	0.016
*Wind*	-0.0019	-0.0437	0.0189
*Temperature*	-0.0941*	-0.1173***	-0.0544
*Humidity*	-0.0507	-0.1050***	-0.0012
*Night light density*	0.0737**	0.0710*	0.1227***

*** p<0.01

** p<0.05

* p<0.1.

A regression model is established with LTPA as the dependent variable, while BE indicators and other potential influencing factors as the independent variables.

$$LTPA=\beta_{1}*Population\;density+\beta_{2}*POI\;density+\beta_{3}*Connectivity+\beta_{4}*Land\;use\;mix+\beta_{5}*Road\;density+\beta_{6}*Park\;and\;square+\beta_{7}*Density\;of\;stadium+\beta_{8}*Total\;population+\beta_{9}*Gender+\beta_{10}*Age\;group+\beta_{11}*GDP\;per\;capita+\beta_{12}*Air\;quality+\beta_{13}*Wind+\beta_{14}*Temperature+\beta_{15}*Humidity+\beta_{16}*Night\;light\;density+\varepsilon$$

where *β*_*i*_ refers to the coefficients of the independent BE variables and control variables, and *ε* is the random disturbance term.

Robust regression has been conducted in Stata to examine the correlations between LTPA and BE variables. Robust regression is an alternative form of regression analysis designed to overcome some limitations of the widely used ordinary least squares (OLS), such as the outliers in data and thus is widely used in social science. In this paper, we employed the least median of square (LMS) method, which requires the estimator to yield the smallest value for the median of squared residuals computed for the entire data set.

The dependent variable, the independent BE variables, including population density, POI density, connectivity, road density and density of parks and squares, as well as some control variables, including total population, density of stadium, and GDP per capita were first taken the natural logarithm to make the data more normally distributed. Also, by doing so, the natural log transformation simplifies the interpretation of the resulting coefficients for BE variables. For example, we can tell the percentage change in LTPA for a fixed percentage change in the BE variable, such as a 10% increase in population density, rather than how many more people per sq. km.

## Results

Three models were established and regressed, and Tables 4 and 5 below list the results. In all three models, the BE variables were taken as natural logarithms to normalize the data distribution and to create interpretations with real-world meanings.

**Table 4:** Regression results of all day LTPA and built environment.

Variables	All day LTPA		
	Coefficient	Beta	95% CI
*Connectivity*	**0.1491** **	0.0930	(0.00, 0.29)
	(0.0738)		
*Population density*	**-0.7873** ***	-0.6408	(-0.94–0.63)
	(0.0780)		
*Road density*	**0.2084** *	0.0822	(-0.01, 0.43)
	(0.1142)		
*POI density*	0.0201	0.0256	(-0.09, 0.13)
	(0.0568)		
*Land use mix*	**1.0520** *	0.1089	(-0.11, 2.11)
	(0.5403)		
*Park and square*	**0.0624** ***	0.0938	(0.02, 0.11)
	(0.0229)		
*Total population*	0.0878***	0.1617	(0.02, 0.15)
	(0.0336)		
*Density of stadium*	-0.0038	-0.0034	(-0.12, 0.11)
	(0.0587)		
*Percent male*	6.3231	0.0605	(-2.59, 15.23)
	(4.5390)		
*Age 65 and older*	7.9265***	0.1711	(3.27, 12.57)
	(2.3744)		
*GDP per capita*	-3.51e-06***	-0.1547	(-5.54e-06, -1.47e-06)
	(1.04e-06)		
*Air quality*	0.0210	0.0217	(-0.06, 0.10)
	(0.0424)		
*Wind*	-0.0043	-0.0353	(-0.01, 0.00)
	(0.0039)		
*Temperature*	-0.0024*	-0.0979	(-0.01, 0.00)
	(0.0013)		
*Humidity*	-0.0009	-0.0132	(-0.01, 0.01)
	(0.0036)		
*Constant*	3.0642		(-2.06, 8.19)
	(2.6101)		
**Observations**	**742**		
**R-squared**	**0.2983**		

Robust standard errors in parentheses.

*** p<0.01

** p<0.05

* p<0.1.

**Table 5:** Regression results of morning and night LTPA and built environment.

Variables	Morning LTPA			Night LTPA		
	Coefficient	Beta	95% CI	Coefficient	Beta	95% CI
*Connectivity*	0.121	0.0733	(-0.04, 0.28)	**0.182** **	0.1092443	(0.03, 0.33)
	(0.0800)			(0.0767)		
*Population density*	**-0.776** ***	-0.6097	(-0.94, -0.62)	**-0.783** ***	-0.6115	(-0.94, -0.62)
	(0.0819)			(0.0815)		
*Road density*	**0.222** *	0.0845	(-0.02, 0.46)	**0.241** **	0.0914	(0.00, 0.48)
	(0.124)			(0.121)		
*POI density*	**0.106** *	0.1305	(-0.01, 0.22)	-0.0218	-0.0267	(-0.13, 0.09)
	(0.0594)			(0.0558)		
*Land use mix*	**1.010** *	0.1009	(-0.10, 2.12)	**0.971** *	0.0967	(-0.03, 1.98)
	(0.565)			(0.513)		
*Park and square*	**0.0662** ***	0.0960	(0.02, 0.11)	**0.0613** ***	0.0886	(0.02, 0.11)
	(0.0240)			(0.0234)		
*Total population*	0.0811**	0.1439	(0.01, 0.15)	0.182**	0.1915	(0.04, 0.17)
	(0.0354)			(0.0767)		
*Density of stadium*	-0.0137	-0.0119	(-0.13, 0.11)	-0.783***	0.0043	(-0.11, 0.12)
	(0.0605)			(0.0815)		
*Percent male*	6.369	0.0588	(-2.71, 15.45)	0.241**	0.0942	(0.33, 20.17)
	(4.625)			(0.121)		
*Age 65 and older*	7.302***	0.1520	(2.56, 12.05)	-0.0218	0.1719	(3.32, 13.25)
	(2.418)			(0.0558)		
*GDP per capita*	-4.42e-06***	-0.1882	(-6.55e-06, -2.29e-06)	0.971*	-0.1187	(-4.82e-06, -7.74e-06)
	(1.09e-06)			(0.513)		
*Air quality*	0.0273	0.0272	(-0.06, 0.12)	0.0613***	0.0091	(-0.08, 0.10)
	(0.0462)			(0.0234)		
*Wind*	-0.00749*	-0.0591	(-0.16, 0.00)	0.108***	-0.0471	(-0.01, 0.00)
	(0.00414)			(0.0329)		
*Average temperature*	-0.00265**	-0.1034	(-0.01, 0.00)	0.00489	-0.0822	(-0.01, 0.00)
	(0.00134)			(0.0600)		
*Humidity*	-0.00608	-0.0823	(-0.01, 0.00)	10.25**	0.2758	(-0.01, 0.01)
	(0.00388)			(5.053)		
*Nightlight density*				8.285***	0.1632	(0.10, 0.37)
				(2.529)		
*Constant*	1.940		(-3.44, 7.32)	-2.80e-06***		(-10.99, 0.68)
	(2.738)			(1.03e-06)		
**Observations**	**742**				**742**	
**R-squared**	**0.283**				**0.271**	

Robust standard errors in parentheses.

*** p<0.01

** p<0.05

* p<0.1.

The results from Tables 4 and 5 show that road density, density of parks and squares, and land use mix are positively associated with LTPA in all models. Additionally, connectivity shows positive coefficients with LTPA in the models of all day and night LTPA but is nonsignificant in the morning time, while POI density only shows positive results in the morning LTPA model. Remarkably, population density is the only variable that presents negative but significant coefficients in all models, indicating that cities with higher population density are associated with a lower frequency of LTPA per capita recorded by the Codoon app. Therefore, the results of the regression model demonstrate vital enabling factors to enhance LTPA: higher connectivity, more open space, lower crowdedness and higher urban vitality.

### Higher connectivity

The density of junctions and roads are widely used indicators in describing the connectivity of a road network. In this study, road density is significantly associated with per capita LTPA in all three models, implying that urban roads play a considerable role in providing adequate chances for continuous outdoor activities such as walking, cycling and jogging, which is consistent with the previous findings. Additionally, connectivity is positively significant with all day LTPA, and the model indicates that a 10% increase of the number of junctions in a city is associated with a 1.49% increase of the LTPA frequency. This is because higher connectivity provides higher accessibility to urban facilities such as sports courts and fitness clubs, which in turn encourages residents to exercise more frequently. Furthermore, higher connectivity is perceived as a BE characteristic that may increase safety from crimes [31] and traffic accidents [32], especially for night runners and children. Our nighttime model supports the argument about safety concerns (see Table 5), as (1) the percentage of males only shows a positive and significant correlation with LTPA at night, indicating that male exercisers are the main population from 18:00 to 21:00; and (2) the positively associated night light density also implies safety as a priority concern for night runners. Overall, a well-organized road network provides more opportunities and guarantees of safety for people, enabling them to engage in more outdoor PA.

### More open space

Similar and expected results are also found in the density of parks and squares, which is significantly associated with per capita LTPA in all three models. Doubling the area of parks and squares in a city would bring an increase of the LTPA by more than 6%. A higher density of parks and squares means more open and public space in the city and more opportunities for residents to engage in outdoor activities at a higher frequency. As mentioned in previous studies, citizens can meet and communicate in urban public spaces such as squares, parks, and plazas, enabling the creation of a diverse urban scene [33]. Within the context of Asian culture, people like to gather and exercise in groups, such as square dances in the morning and evening. Such preference also attracts more people and aggregates into a kind of atmosphere of exercise. Thus, open space plays a vital role in enhancing the overall level of LTPA for urban life.

### Lower crowdedness

The results of these models showed an inverse association of population density with LTPA, implying a decrease of LTPA by nearly 8% for every 10% increase of population density. The population density has historically been thought to exert positive effects on encouraging PA, since higher density is generally associated with smaller blocks, more mixed land use and shorter distance to destinations [34]. However, higher population density alone does not appear to be a proven factor for increased LTPA, as least as shown in this study. Conversely, a high population density also indicates a high level of crowdedness and busy traffic. Xu et al. [35] found the same correlation and their explanations have convinced us that high density scenarios in Chinese cities might be different from other countries, which results in contrary correlations between LTPA and population density. They also suggested that densely settled Chinese cities could hinder LTPA due to decreased availability of PA resources and increased concerns about traffic safety. In addition, we also found that Wang et al. [36] in their empirical analysis for Chinese cities showed participants lived in communities with higher and middle residential density were significantly less likely to achieve sufficient physical activity relative to their counterparts lived in the lower densed communities. They also mentioned that their findings are inconsistent with the majority of literature from Western countries, but in line with their previous study conducted among urban Chinese adolescents in the same city, and they attribute such results to the potential inhibitor to outdoor physical activity caused by higher roads/streets and traffic volume around neighborhoods, which also supported our judgement.

### Higher vitality

In this study, POI density and land use mix represent the level of urban vitality since the higher mixture of functions and higher diversity of interests and attractions in the city could be on behalf of the high vitality of urbanization. Land use mix exhibited significant results in all models at the p<0.1 level. The positive coefficients indicate that a balanced mix of different urban functions in a city exerts positive influence on LTPA behaviors. For indoor LTPA, such as swimming or gym fitness, higher POI density and land use mix indicates more chances to find such facilities near home or workplace, which encourage people to participate in more LTPA. While for outdoor activities, such as walking and jogging, higher vitality is associated with safer environment, which is a major concern for urban sportsman. Although POI density only shows a significant correlation with LTPA in the morning session, the positive coefficient implies that cities with higher POI density may encourage residents to take morning exercise more often. Most groceries and shops open at 6 am and close, approximately, between 8 pm and 9 pm. This timeframe also explains why POI density has less influence on nighttime exercise but shows positive effects on morning exercise, since morning exercise may also relate to the traditional morning market in China, where people have breakfast and engage in simple grocery shopping.

To compare the effects of different BE variables on LTPA, the standardized coefficients (Beta) are also calculated in each model. The Beta coefficients in the regression results also tell us which BE variable has a greater effect on the level of LTPA. For example, all three models imply that population density has greater effects on LTPA than any other BE variables. Larger Beta coefficient of population density indicates that for every increase of one standard deviation in all BE variables, population density would be associated with more change of LTPA in terms of standard deviation. However, it does not indicate that decreasing the population density could be the most effective way to encourage LTPA, and we should notice that it takes much more efforts and longer time to change population density than changing other BE indicators, such as doubling the areas of parks or developing more POIs. While the models could help predicting the changes of LTPA under different planning scenarios. For BE variables that exert positive effects on LTPA, land use mix has a stronger influence than connectivity for all day activities, but the influence is weaker at night, as the closing of stores and office buildings weakens the mix of urban function. As stated above, connectivity as a safety-related attribute exerts stronger impact on night LTPA.

## Discussion

Taking Chinese cities as examples, the present study empirically examined the correlations between LTPA and BE, using the massive LTPA data generated by Codoon, a popular fitness app. Five of six BE attributes—connectivity, road density, land use mix, POI density, and density of parks and squares—were significantly, positively, independently, and linearly related to LTPA in the regression analysis. Our results for connectivity, land use mix, road density, and density of parks and squares are consistent with the previous findings in the literature [4–6, 37]. Moreover, this study provides novel insights into the important role of POI density in encouraging LTPA, especially when Chinese traditions and culture are considered, as few studies have considered POI density as one influential BE factor in LTPA. Population density shows negative effects on LTPA, which, although has no unifying conclusion in previous studies, can also be explained in the Chinese context [35]. This study also sheds light on how LTPA at different times of the day is affected by different BE attributes, particularly for POI density in the morning and connectivity at night. Improved evidence on the relationship between BE and LTPA is crucial, as environments are constantly changing in ways that could have positive or negative effects on whole populations over many years. The present findings support the recommendation that densely distributed and interconnected streets, mixed land use, a higher level of POI density and accessibility to parks and squares should be prioritized when designing PA-supportive environments.

The added value of this paper includes the following: (1) new technology and new data have been proven to have great potential in answering some classical issues; (2) an analysis of nationwide data was designed to improve the quality of evidence by examining a broader range of BE and LTPA behaviors with a much larger sample size; (3) comparable objective measures of BE and LTPA are proposed that can be duplicated worldwide; (4) the behavior of engaging in LTPA varies at different times of the day, as do BE and the correlation between them; and (5) the findings of this study advocate collaboration between public health proponents and other sectors, including urban planning authorities, to promote PA-supportive development.

Several limitations are notable as well. First, although Codoon app has a large share in Chinese users, indicating a good sample for national analysis, there are still other popular activity app, such as Keep, Yund that have large user groups, which might cause potential bias to our dataset due to the users’ self-selection. Second, some data, such has the Landscan population data is from the year of 2016, due to the availability of data when the analysis was conducted. Road density is used as the best proxy of sidewalks and bike lanes, as these GIS data are quite limited in China. Third, the frequency of LTPA reflects only one basic aspect of PA, while activity type and duration are not included at this stage of the study. In addition, educational attainment [17, 38] and employment status [18] have all been found to moderate associations with PA, albeit inconsistently, however, they are not included as control variables in the paper due to data limitations for redefined natural cities.

LTPA, especially outdoor activities, may exhibit seasonal patterns. Although the Codoon database includes the LTPA from January to June in 2018, they were aggregated to a cross-sectional dataset due to privacy reasons, which weakens the seasonal fluctuations, as well as the influences of temperature, wind speed and humidity, as these were calculated as averaged across 6 months.

Admittedly, an individual study cannot make the fullest use of the massive amount of data provided by Codoon. With such fine-scale LTPA data, we are able to examine how intracity- and neighborhood-level BE factors are related to PA, as well as how these patterns might shift between seasons in our future studies. In addition, Codoon has more than 100 million registered users in 210 countries worldwide. The interaction effects between or among BE variables could be tested with existing data to examine whether the expected influence from higher density is dependent on other BE indicators. More LTPA-related BE variables could be included in the regression analysis if data become available, such as the density of sidewalks, bike lanes and facilities, and factors affecting road intersection quality, such as crosswalks, overpasses or underground passages. Furthermore, prospective studies and quasi-experimental evaluations in applicable locations could be conducted to isolate the influences from BE.

## Supporting information

S1 File Anonymized Codoon data at city level. (DTA)
